# The Alberta Back Care Pathway: The feasibility of implementing a novel care pathway to improve low back pain management for family physicians in primary care

**DOI:** 10.1371/journal.pone.0312737

**Published:** 2024-11-27

**Authors:** Brandyn Powelske, Alice Kongsted, Allyson Jones, Gregory Kawchuk

**Affiliations:** 1 Faculty of Rehabilitation Medicine, Department of Physical Therapy, University of Alberta, Edmonton, Canada; 2 Department of Sport Science and Clinical Biomechanics, University of Southern Denmark, Odense, Denmark; University of Limpopo, SOUTH AFRICA

## Abstract

**Background:**

Family physicians in Canada’s universal healthcare system often manage low back pain patients using interventions not recommended in clinical guidelines, such as pharmaceuticals, imaging and spinal injections, while guideline-based treatments like education and exercise remain unfunded. The Alberta Back Care pathway was developed to address this gap, offering funded, evidence-based care for low back pain patients in 5 streams (acute, sub-acute, chronic, chronic non-responsive and stable radiculopathy).

**Objective:**

To evaluate the feasibility of implementing the pathway in two urban Primary Care Networks in Alberta, Canada.

**Materials and methods:**

Each of the 5 pathway streams provided physicians with information scripts, no-cost interventions (pharmaceuticals and otherwise) and interventions to avoid. From April 2021 to November 2023, the RE-AIM framework was used to assess implementation feasibility of the pathway.

**Results:**

For the RE-AIM dimension of reach, 25% (n = 41/162) of eligible family physicians in Primary Care Network “A” and 12% (n = 26/221) in Primary Care Network “B” enrolled in the study. Over half of enrolled physicians (n = 21/41 and 21/26) referred at least one patient with most referrals to the GLA:D Back program for chronic low back pain stream (93% in network “A” and 88% in network “B”). Implementation, evaluated as the proportion of referrals by physician compared to their total low back pain caseload, was low (> 0–10% referred) for 52% (n = 11/21) of physicians in network “A”, and medium-low (10–25% referred) for 52% (n = 11/21) of physicians in network “B”. The number of pathway-appropriate patients in each physician’s caseload was unknown. Maintenance at 12 months was 56% (n = 10/18) in network “A” and 39% (n = 7/18) in network “B”.

**Conclusion:**

The Alberta Back Care pathway was feasible to implement during the pandemic and primarily serving patients with chronic low back pain by providing access to a guideline-based education and exercise group program (GLA:D Back).

## Introduction

Globally, low back pain (LBP) has remained the leading cause of disability over the last two decades [1,2]. Serious spinal pathologies like fractures, infections, cauda equina syndrome, and malignancy are rare in those presenting with LBP in primary care [3]. However, LBP can significantly negatively impact a person’s daily activity, participation in work and personal life, and well-being, resulting in lost productivity and excessive healthcare system costs [4]. Given that LBP is responsible for more years lived with disability, 83 million DALYs globally [5], than any other musculoskeletal condition [2,3], it is one of the most common reasons to seek care from a healthcare provider [6,7]. Specifically, 65% of LBP cases in North America are initially assessed and provided an intervention by primary care physicians [8]. In Canada, access to family physicians (FPs) is provided through public funding within a universal healthcare model. Physicians may choose to provide various interventions and services that are not included in most international guidelines for LBP, including pharmaceutical management such as acetaminophen, opioids, and anti-depressant medications; referrals for imaging and special consultations; and spinal injections. Interventions recommended by these same guidelines, such as patient education and therapeutic exercise, are not offered to patients presently in Canada’s universal healthcare system [7,9–13].

Although LBP guidelines are taught in the curriculum for health profession programs and promoted by healthcare practitioner regulatory bodies, LBP guidelines are infrequently implemented by healthcare professionals in primary care [14]. This is due to well-recognized barriers such as a lack of FP training or confidence [15]; overall clinical impracticality of delivering guideline-based interventions, including time and financial compensation [16]; and lack of patient funding for guideline-based interventions that require out-of-pocket expenses for their patients [17,18].

Well-defined and financially supported clinical pathways may serve as a tool to support and facilitate FP adherence to clinical practice guidelines. In this context, clinical pathways suggest a practical application of the guidelines. Clinical care pathways facilitate communication between healthcare practitioners and their patients, coordinate care through a planned sequence of activities, continuously monitor the pathway through outcomes, and allocate appropriate resources for the pathway to function [19]. It is, therefore, possible for the evaluation of clinical pathways to contribute to the ongoing process of improving evidence-based care for patients in terms of quality and efficiency [20].

In this context, the Alberta Back Care Pathway (ABCp) was developed to support FPs in delivering funded guideline-based care for patients seeking medical attention for LBP. For each of the five pathway streams (acute, sub-acute, chronic, non-responsive chronic, and stable radiculopathy), ABCp was designed to provide FPs with prognostic information, at least one fully funded evidence-based clinical treatment option, and a list of non-guideline procedures to avoid. These options included both pharmacological and non-pharmacological LBP treatment options. One of the conservative management options included GLA:D Back, which is a group-based program consisting of education and exercise specifically for LBP. While previously developed pathways have demonstrated evidence for the reduction in wait times for LBP surgical consultations, decreased referring MRI use, and improved appropriate referrals to spine specialists [21,22], there has been limited implementation of pathways that have incorporated funded, evidence-based care for the conservative management of LBP for patients that are not appropriate to receive care from specialists. Often, these patients are left with limited treatment options or fragmented care to manage their LBP.

The ABCp is aligned with the key goals outlined by Health Canada’s Action Plan for Pain in Canada 2021 Report, which includes: 1) improving access to timely, equitable, and person-centred pain care, 2) increasing both people with pain and health professional’s awareness, education, and specialized training for pain, 3) supporting pain research and related infrastructures to enable discovery, innovation and results in the translation of knowledge into real world impact, 4) monitor population health and health system quality through data, and 5) ensuring equitable approaches for populations disproportionally impacted by pain [23].

The overall aim of this study was to assess how the ABCp was used by FP and their patients managing LBP and both FP and patient-related outcomes of using the pathway. Given this, the primary objective of this study was to evaluate the feasibility of implementing the ABCp at two urban Primary Care Networks (PCNs) in Alberta, Canada, during the pandemic, and using the RE-AIM framework.

## Materials and methods

### Design and settings

Guided by a type two hybrid implementation effectiveness study design [24], this feasibility study evaluated the implementation of the ABCp in two large urban PCNs in Alberta, Canada, from April 2021 to November 2023. PCNs are partnerships between publicly funded provincial healthcare services (Alberta Health Services) and FPs in a similar geographic area that provide interdisciplinary care with other allied healthcare practitioners, including nurse practitioners, registered dietitians, and exercise specialists. There are 39 PCNs in Alberta, with 22 designated as rural and 17 as urban. The number of FPs contracted by Alberta PCNs is approximately 3800, and there are over 1400 full-time, part-time, and casual other allied healthcare staff serving around 3.6 million Albertans.

This study assessed the feasibility of implementing the ABCp in the first two PCNs that agreed to run the pathway. PCN A serves approximately 182,000 patients in a large urban community supported by the care of 100 health professionals and over 180 physicians. PCN B also serves a large urban community where over 230 physicians and 110 health professionals provide care to 193,000 patients. Evaluating feasibility in two similar PCNs was used as a simple strategy to compare implementation results in similar settings. Considering that both PCNs are in the same urban geographical setting, with similar numbers of staff, and serve similar patient populations, we hypothesized that the outcomes of the feasibility of implementation would be comparable between the two.

This study was registered in clinicaltrials.gov on July 11, 2022 (Identifier: NCT05452876). Research ethics approval was granted by the University of Alberta Human Research Ethics Board (Pro00105576).

### Family physician participants

**Fig 1:**
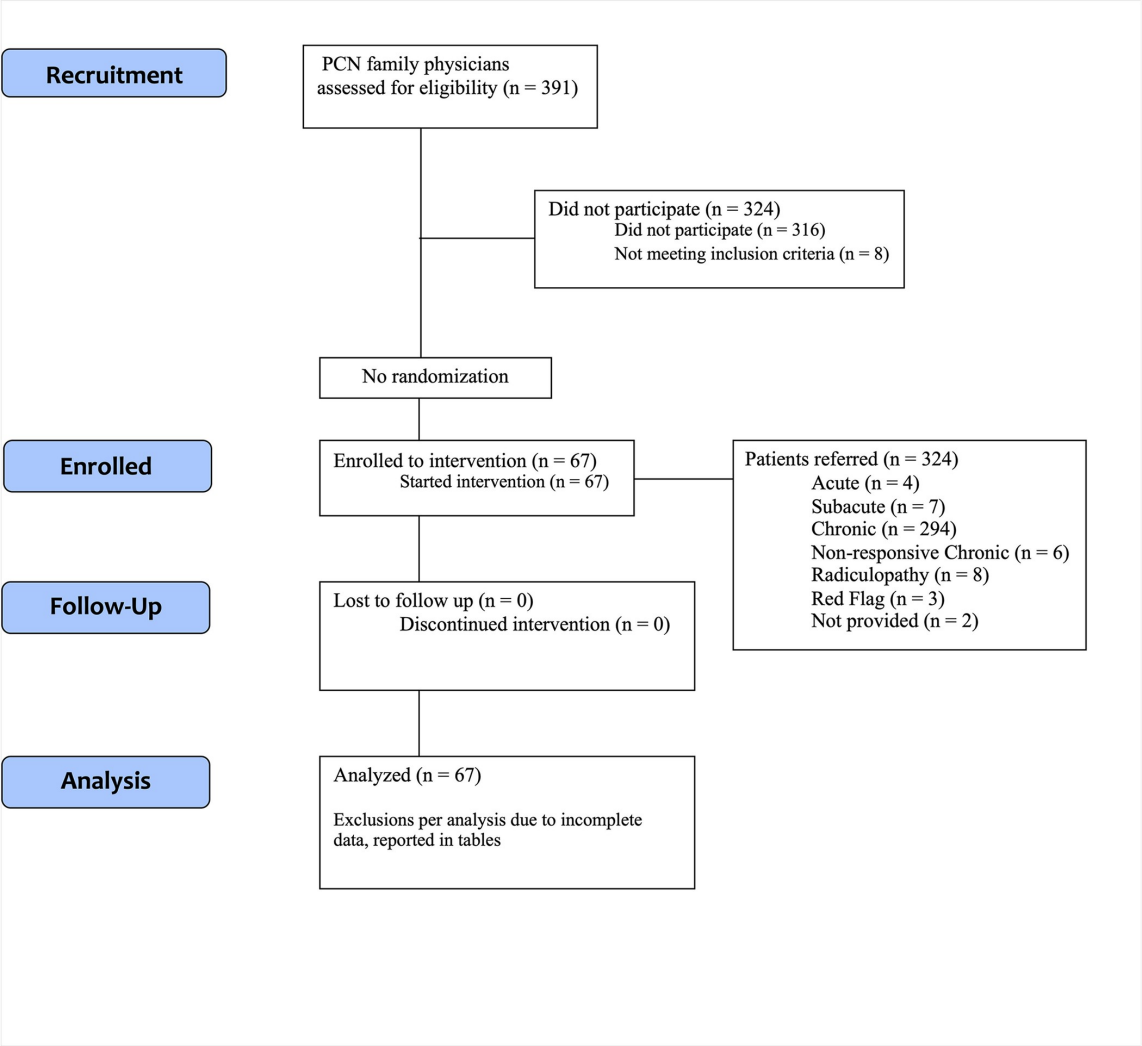
CONSORT flow diagram for PCN A and B.

In this study the feasibility of implementing the ABCp was assessed through FPs working in two PCNs. Family physicians were the focus because they provide the majority of care for LBP patients, largely because in our jurisdiction, they can provide that care at no cost to patients yet lack access to guideline-based care. Addressing this gap through an easy-to-implement, no-cost intervention could significantly impact both patient outcomes and healthcare resource use. Accordingly, this study has two types of participants: FPs and their patients. Neither group was blinded to the intervention. Each PCN was asked to identify FPs with special interests, like pediatrics and women’s health, who would not be included in the target group for the study. Those FPs from PCN A and PCN B who met the inclusion criteria were invited to participate in the study. Due to the complexity and wide variation in the clinical physician-patient interaction, this study was designed to be flexible and accommodating for FP participants and their patients. By considering the variability in physician-patient interactions in the study design, the research team felt that the results are representative of implementing a care pathway in real-world clinical practice. The flow of participants from PCN A and B through each stage of the study is summarized in the CONSORT diagram (Fig 1). Once an FP was enrolled and completed the online training, they were eligible to utilize the pathway for any adult patient with LBP in their caseload. A rolling recruitment of FPs was done throughout the 12-month observation period of the study, and therefore no strategies such as matching were used. Given the pragmatic design of this feasibility study to understand implementation of the pathway, no formal sample size was calculated.

### The Alberta Back Care Pathway intervention and patient participants

**Fig 2:**
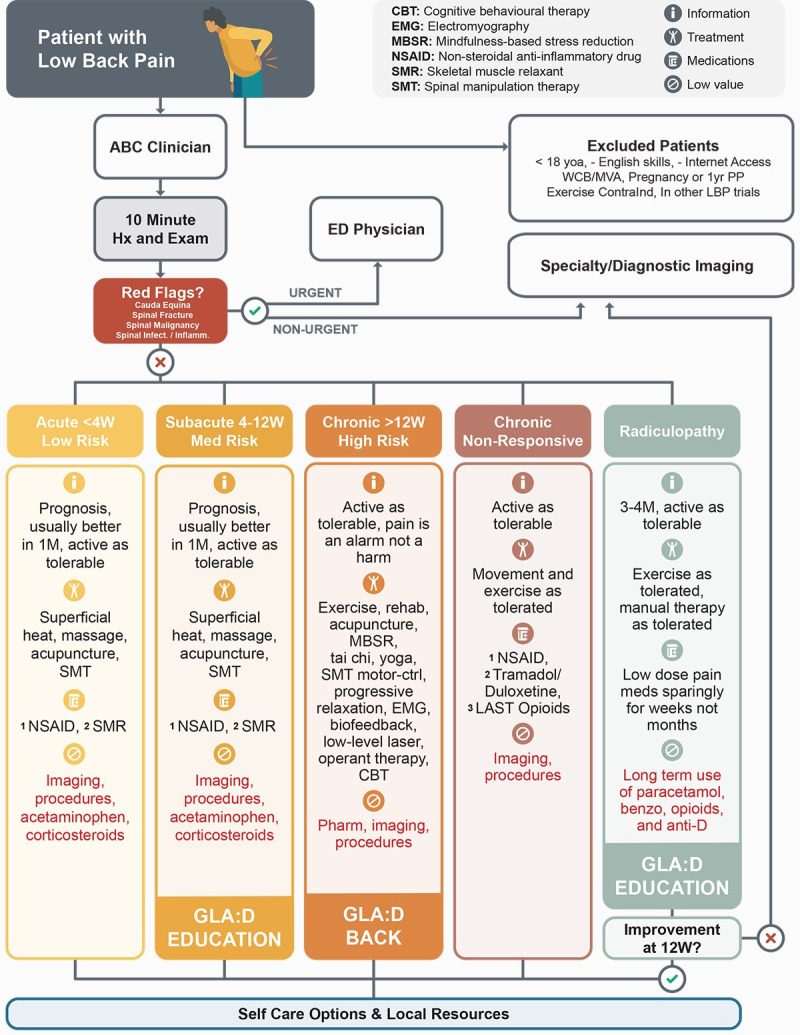
The Alberta Back Care Pathway.

The ABCp was co-designed by administrators, researchers, physicians, and patients based on the recommendations of the American College of Physicians and Danish Health Authority LBP clinical practice guidelines [7,10]. These guidelines provide specific recommendations for clinical interventions related to acute, sub-acute, chronic, non-responsive chronic, and stable radicular LBP. For each of these five LBP categories, the ABCp translated these guidelines into recommended care pathway streams for FPs by providing specific information regarding prognosis, recommending non-pharmacological interventions (with at least one funded treatment), and providing possible pharmacological interventions, and a list of interventions and procedures that are not supported by the evidence (Fig 2). Suspected serious lumbar pathologies, “Red Flags”, were screened by the FP, and these patients were referred to the emergency department, diagnostic imaging, or a specialist as deemed necessary by the FP. Three of the five streams included education and/or exercise provided by the GLA:D Back program (see below).

After evaluating the patient’s complaint, history, physical exam, and triaging for serious lumbar pathologies, FPs could refer their patient to one of ABCp’s five streams (acute, sub-acute, chronic, chronic non-responsive, and stable radiculopathy) to utilize the pathway. Once the FP identified the ABCp LBP category for the patient and if the patient consented to participate, a research team study coordinator contacted the patient to explain the study, review exclusion criteria, and enroll them. Eligible patients were Alberta residents who were 18 years of age or older, could speak and read English proficiently, and had access to the Internet. Those excluded from the study were persons having an active LBP Workers Compensation Board or motor vehicle accident claim, had recent spinal surgery, or were enrolled in another clinical trial. The clinicians who delivered the GLA:D Back program additionally screened each patient to see if they had any contraindications to exercise and if they were appropriate for the program. Patients not choosing to enroll and those not referred to the pathway were offered usual care by the FP (which did not include the GLA:D Back program).

#### Family physician training

As part of the implementation plan, FP training on the ABCp was provided through a self-paced online course (Articulate, New York, USA), which took approximately one hour to complete. The training course was developed by members of the research team with the goal that the participating FP should be able to categorize their patient into the five ABCp LBP classifications, provide the suggested intervention/s, and understand the administrative process of the pathway. The ABCp training consisted of nine modules: 1) ABCp Overview and Inclusion/Exclusion Criteria, 2) LBP Red Flags, 3) Ten-minute LBP History and Examination, 4) Acute LBP, 5) Subacute LBP, 6) Chronic LBP, 7) Chronic Non-responsive LBP, 8) Lumbar Radiculopathy, and 9) What is GLA:D Back? and Clinical Administration. Interactive components included case studies designed to stimulate participant review and reflect on key messages in each module. Each module included a skills check, which served as a self-assessment exercise without a pass or fail rating. The training course was left open to the FP to access at any time if they wanted to reference it in the future. FPs could apply to use the course as a credit part of the Alberta College of Physicians continuing medical education program.

#### GLA:D Back program

Developed in Denmark, GLA:D Back is an evidence-based program designed to promote self-management, self-efficacy, and improve overall well-being and quality of life for those living with LBP [25]. GLA:D Back was implemented in Demark in 2017 [26] where 808 clinicians from 384 clinics participated in the clinical courses by the end of 2023 [27]. Patient outcomes of the program include decreased medication use, less fear of physical activity, and increased physical function after completing the multi-week program [28]. In 2019, the GLA:D Back program was translated from Danish to English and piloted in Alberta, Canada. This version of the GLA:D Back program was found feasible to implement by physical therapists and chiropractors working in private and publicly funded clinical settings [29]. The feasibility of implementing GLA:D Back has been tested in multiple healthcare settings, most recently in Australia [30]. GLA:D Back, introduced as part of the ABCp, is a new offer not otherwise available in public health care in Alberta.

As described in more detail previously [31], the GLA:D Back program is comprised of structured, group-based education and exercise sessions led by healthcare providers trained to deliver the program in a 2-day training course. At the program’s start, individuals’ baselines are assessed one-on-one using standardized objective tests, and patient-specific goals are established using the SMART goal format. Once the baseline is completed, the participants attend two one-hour education sessions followed by 16 one-hour exercise sessions twice weekly. These sessions are group-based, with a recommended number of participants between 4–10. Throughout the program, education and exercise are combined to build self-management strategies for participants [25]. Eight key messages explaining LBP as a non-threatening condition and promoting physical activity are highlighted throughout the program.

The GLA:D Back program in Canada typically requires patient payment; however, as part of the ABCp, PCN exercise specialists were trained to provide GLA:D Back as part of their salaried duties.

### Implementation strategy

Overall guidance for planning and support of the implementation process was motivated by the Quality Implementation Framework (QIF) [32], which consists of four phases to guide implementation: initial considerations assessment of the host setting, creating a structure for implementation, ongoing implementation support, and learning from experience to improve future applications. Each phase was adapted, tailored, and modified to meet the individual needs of each PCN.

In brief, our implementation strategy included one-on-one contact with PCN staff using Zoom to foster a relationship, understand the differences between each PCN’s organizational capacity characteristics, and assess the overall PCN resources available to implement ABCp. At each PCN, communication outlets for delivering ABCp information for FPs and other PCN staff included digital multimedia, info-graphic posters and handouts, videos, and newsletters delivered through email and websites. These communications included an overview of the ABCp, essential information on the pathway for FPs, and instructions on enrolling patients in the study. FPs and leadership team engagement opportunities were also offered as opportunities for clinicians and other PCN staff to learn and ask questions about the ABCp in-person or online. The ABCp research team was available by email and phone to address any questions or concerns raised by the PCNs. Last, FP and other PCN staff feedback was continuously incorporated as part of a Plan-Do-Study-Act cycle approach. This cyclic process of reflecting on the lessons learned and experiences from the implementation at each PCN allowed us to continuously adapt the implementation process to the needs of each individual PCN, their staff, and FP members.

### Data collection and outcomes

#### Family physician data

Electronic surveys were sent to FPs at two time points: at their enrollment and four months after the baseline survey was completed. RedCap (Research Electronic Data Capture) was used to collect electronic questionnaire responses from FPs.

The baseline survey included questions on clinician demographics, including age, sex, clinical education, years of clinical experience, terms of employment at the workplace (e.g., clinical owner, employee, or contractor), and practice location. Baseline survey data also included FP confidence in managing patients with LBP and their attitudes and beliefs about back pain. Confidence in providing care for LBP patients was assessed by the Practitioner Confidence Scale (PCS). The PCS contains four items using a five-point Likert scale that evaluates clinician confidence in managing LBP. A summative score is generated ranging from 4 to 20, where lower scores indicate higher confidence [33]. Clinician attitudes and beliefs about back pain were evaluated using the Pain Attitudes and Beliefs Scale (PABS). The 19-item PABS rated on a 6-point Likert scale is made up of two domains, biomedical (sum score 10–60) and biopsychosocial (sum score 9–54), where a high score represents a higher orientation to that domain [34]. The PCS and PABS were sent in baseline surveys and were collected as secondary measurements, with implementation outcomes described below being the primary.

In the 4-month follow-up survey, FPs were asked about: their satisfaction with the ABCp using a five-point Likert scale (1 = “very dissatisfied” to 5 “very satisfied”), how well the pathway worked for them in clinical practice (1 = “very bad” to 5 “very good”), and the most common reasons their patients did not want to be referred to the pathway (cost, time, did not want guided exercise or education training, too comprehensive for their problem, or other). The Determinants of Implementation Behavior Questionnaire (DIBQ) was also included in the follow-up survey which consists of 31 questions organized into ten domains that include knowledge, skills, beliefs about capability, beliefs about consequences, innovation, organization, patients, intentions, social influence, behavioral regulation [35]. Similar to a study that evaluated the implementation behavior of clinicians implementing a LBP program [36], the 5-point DIBQ Likert scale (1 = “strongly agree” to 5 = “strongly disagree”) score in our study was also transformed to a percentage (100% = “strongly agree” to 0% = “strongly disagree”) where a score over 50% suggests an implementation facilitator. The PCS and PABS were also included in the follow-up survey to determine if confidence and attitudes/beliefs about back pain had changed since baseline.

The number of unique LBP patient encounters by each FP was gathered from the practitioner claims data source via approved access to the Alberta Health Services Enterprise Data Warehouse data repository from April 22, 2020, to August 31, 2023.

#### Implementation outcomes

Using the RE-AIM framework [37], the ABCp implementation was evaluated at the individual FP level (Table 1). Based on the previous GLA:D Back Alberta feasibility study [29], our criteria for feasibility implementation success was predefined at 50% for each of the dimensions of reach, adoption, implementation, and maintenance.

**Table 1:** RE-AIM dimensions definitions and measurements [38,39].

RE-AIM Dimension	Definition	How dimension was measured in this study
**Reach **	The proportion of individuals of the targeted population that is impacted by a particular initiative, intervention, or program.	Measured by the percent of individual FPs who participated in the pathway out of the potential eligible FPs at the PCN.
**Effectiveness **	The impact on health, mental health, or other individual-level primary outcomes, including positive, negative, and unintended consequences.	Dimension not reported in this paper.
**Adoption **	The number of settings and providers willing not only to participate but also utilize the initiative, intervention, or program.	The proportion of FPs who utilized the pathway in clinical practice out of the total number of FPs enrolled in ABCp. Utilization of the ABCp was defined in this study as an FP applied a low back ABCp category and referred at least one patient to the ABCp.
**Implementation **	The ability of participants fidelity to the various elements of an intervention’s key functions or components, including consistency of delivery as intended and the time and cost of the intervention. Importantly, it also includes adaptations made to interventions and implementation strategies.	Assessed as the proportion of patients with LBP referred to the ABCp. The referral proportion (%) for each participating FP was calculated by the total number of LBP patients referred to the ABCp divided by the total unique LBP patients seen by the FP in clinical practice over the study period. FP adherence rate was defined categorically as Low (>1–10%), Medium-Low (10–20%), Medium (20–50%) and High (>50%).
**Maintenance **	The degree to which the program is sustained and to which the effects of the program are maintained.	The degree to which FP use of ABCp was sustained over 3-6-12 months after implementation was first initiated. The percent of FP that continued to refer at least one new LBP patient to ABCp in the 3^rd^, 6^th^ and 12th months after referring their first patient to the pathway.

### Statistical analysis

A descriptive statistical analysis was performed and presented for all quantitative data collected, including FP baseline demographics and measurement of implementation outcomes based on the domains within the evaluation RE-AIM framework, with the exception of effectiveness. R software (Version 4.3) was used to analyze descriptive statistics for the baseline FP characteristics, 4-month survey outcomes, and implementation outcome, including n (%), mean (sd), and median (IQR or range) as appropriate. Missing data for participant-reported outcome measures were addressed using a complete-case analysis approach. Given that this was a feasibility of implementation study, no inferential statistics were performed.

## Results

### Family physician demographics at baseline

PCN A was the first to implement the ABCp, with 41 FPs enrolled in the study. Only four (9.8%) FPs completed the physician ABCp training before utilizing the ABCp. FPs were (46)% female and had a mean age of 46 years (sd = 12). The clinic employment structure was 27% of FPs being clinical owners, 17% clinic employees, 46% self-employed or contractors and 2% listed as “other”. Practice experience varied among participating FPs. However, the majority (59%) had over ten years of experience in clinical practice. Both FPs who completed the FP ABCp training modules and those who did not had moderate confidence in treating patients with LBP (PCS score mean ± sd = 11.2 ±1.9) and 95% (n = 36/38) had a higher biopsychosocial (mean ± sd = 41.5 ± 4.1) than biomedical score (mean ± sd = 30.5 ± 5.2) on the PABS (Table 2).

**Table 2:** Family physician baseline characteristics and select outcomes.

		n (%); Mean ± sd
	N = 41	N = 26
**PCN**	A	B
**Number of years of clinical experience**		
0–5 years	7 (17%)	2 (7.7%)
6–10 years	8 (20%)	5 (19%)
11–15 years	8 (20%)	8 (31%)
16–20 years	2 (4.9%)	3 (12%)
Over 20 years	14 (34%)	8 (31%)
Incomplete	2	
**Sex**		
Female	19 (46%)	17 (65%)
Male	21 (51%)	9 (35%)
Incomplete	1	
**Terms of employment**		
Clinic Owner	11 (27%)	3 (12%)
Clinic Employee	7 (17%)	6 (23%)
Self-employed	19 (46%)	14 (54%)
Other	1 (2.4%)	2 (7.7%)
Incomplete	3	1
**Age**	46 ± 12	46 ± 9
Incomplete		1
**Baseline PCS Score**	11.2 ± 1.9	11.2 ± 1.5
Incomplete		1
**Baseline PABS Biomedical**	30.6 ± 5.2	28.6 ± 6.6
Incomplete		1
**Baseline PABS Biopsychosocial**	41.4 ± 4.1	39.7 ± 4.7
Incomplete		1

Of the twenty-six FPs from PCN B who enrolled in ABCp, only two completed the physician ABCp training, representing a completion rate of 7.7%. FPs were 65% female and had a mean age of 46 years (sd = 9). Like in PCN A, most FPs were self-employed or contractors (54%), with the rest split between 12% being clinical owners, 23% employees of a clinic, and 8% listed as “other”. Practice experience also varied, with most FPs being senior practitioners with over ten years of experience (73%). Family physicians had moderate confidence in treating and managing patients with LBP at baseline (PCS score mean ± sd = 11.2 ±1.5) and 96% (n = 24/25) of FPs had a higher biopsychosocial (mean ± sd = 39.7 ± 4.7) than biomedical score (mean ± sd = 28.6 ± 6.6) on the PABS (Table 2).

### Family physician 4-month survey results

Response rates on the 4-month survey were low at PCN A, with only 7/41 (17%) of the enrolled FPs completing the survey. Of the FPs that completed the survey most 5/7 (71%) reported ABCp worked well for them in clinical practice, and 4/7 (57%) FPs reported time as the most common reason their patients did not want to be referred to ABCp. Within this group there was high satisfaction of the overall ABCp itself by FPs with 6/7 (86%) reporting “satisfied” (Table 3).

**Table 3:** Family physician 4-month survey outcomes.

		n (%); Median (Range)
4-survey response	N = 7/41	N = 6/26
**PCN**	A	B
**Most common reason FP reported why patients did not want to participate in ABCp**		
Cost	0 (0%)	1 (17%)
Time	4 (57%)	3 (50%)
Too comprehensive	1 (14%)	0 (0%)
Did not want guided exercise training	2 (29%)	1 (17%)
Did not want education training	1 (14%)	1 (17%)
Other	2 (29%)	1 (17%)
**Overall satisfaction with of ABCp**		
Satisfied or Highly Satisfied	6 (86%)	4 (67%)
Neutral	1 (14%)	2 (33%)
Not Satisfied or Highly Not Satisfied	0 (0%)	0 (0%)
**4 Month PCS Score**	8 (8–10)	8.5 (4–12)
**4 Month PABS Biomedical**	34 (20–45)	26 (21–33)
**4 Month PABS Biopsychosocial**	44 (42–48)	38.5 (36–42)
**DIBP-Q (%)**	62.5 (53.3–68.3)	60.4 (49.2–82.5)

At PCN B the response rate was also low with 6/26 (23%) of the enrolled FPs completing the 4-month follow-up survey. Most 4/6 (67%) of FPs who completed the survey reported ABCp worked well or very well in their clinical practice. Like in PCN A, the most common reason reported by FPs, 4/6 (67%), was time for their patient not wanting to be referred to the pathway. Similar to what was observed at PCN A, PCN B also reported high satisfaction with the overall ABCp itself with 4/6 (67%) FPs reporting “satisfied” or “highly satisfied” (Table 3).

### ABCp LBP category referral results

**Fig 3:**
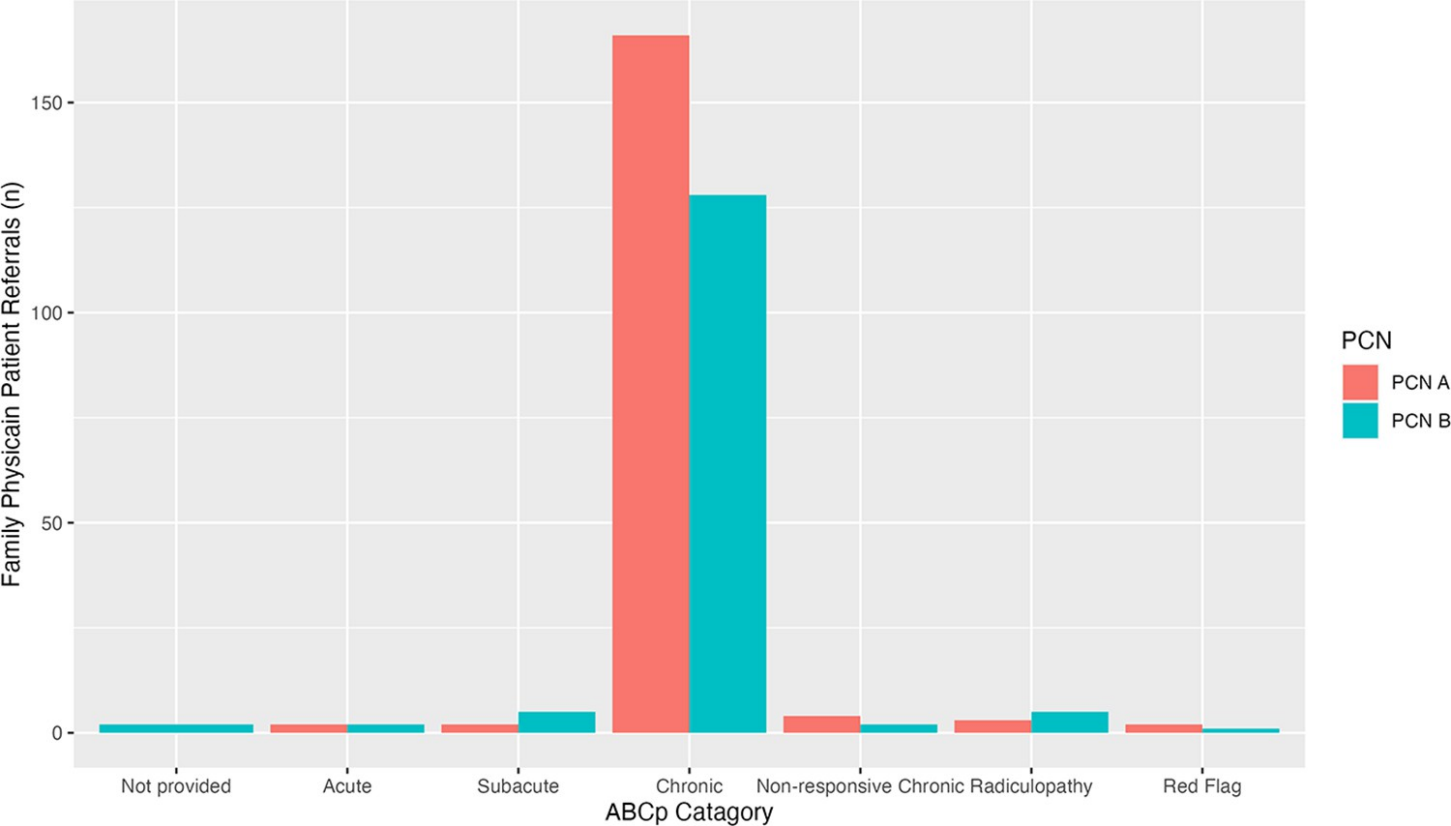
Referred patients to the Alberta Back Care Pathway categories.

In PCN A and B, the ABCp was mainly used for chronic LBP: 93% and 88% of patients, respectively (Fig 3).

### Implementation outcomes

Implementation outcomes for PCN A and PCN B are summarized in Table 4.

**Table 4:** Family physician RE-AIM results.

RE-AIM Dimension	PCN A	PCN B	
**Reach **	Exclusion criteria (not eligible specialists, etc.): 0/162 (0%)	Exclusion criteria (not eligible specialists, etc.): 8/229 (0.03%)	
	Individual family FPs who enrolled: 41/162 = 25% of potential FPs	Individual family FPs who enrolled: 26/221 = 12% of potential FPs	
**Adoption **	The proportion of FPs who utilized the program: 21/41 = 51%	The proportion of FPs who utilized the program: 21/26 = 81%	
**Implementation**			
	**Variable**	**PCN A**, N = 21^*1*^	**PCN B**, N = 21^*1*^
	**FP Patient Referrals, median (Q1, Q3)**	3 (1, 7)	4 (2, 6)
	**FP Referring Proportion, median (Q1, Q3)**	7 (3, 21)	13 (4, 18)
	**FP Referral Proportion Categories, n (%)**		
	>0–10% (Low)	11 (52%)	8 (38%)
	10–25% (Medium-Low)	5 (24%)	11 (52%)
	25–50% (Medium)	3 (14%)	1 (4.8%)
	>50% (High)	2 (9.5%)	1 (4.8%)
**Maintenance **	FPs referring at least one new patient: ≥ 3 months: 14/21 (67%) ≥ 6 months: 10/21 (48%) ≥ 1 year: 10/18^1^ (56%)	FPs referring at least one new patient: ≥ 3 months: 14/21 (67%) ≥ 6 months: 9/21 (43%) ≥ 1 year: 7/18^1^ (39%)		

^*1*^Three FPs were not enrolled in ABCp for ≥ 1 year.

#### Reach

PCN A had 162 eligible FPs, out of which 41 (25%) enrolled in ABCp. Notably, the initial enrollment rate was low, with only four FPs enrolling in the first four weeks after the ABCp was launched. Subsequently, an increase in FP enrollment was observed in 2022, with the highest rate occurring in September 2022, when eight FPs enrolled for the pathway. In comparison, PCN B had 221 eligible FPs, with eight potential candidates excluded as they were specialists or would not have adult LBP patients on their caseloads, like pediatricians. Enrollment grew steadily, reaching an average of three FPs per month after the first six months with the highest rate occurring in January 2022. In total, 26/221 (12%) FPs enrolled in ABCp at PCN B.

#### Adoption

Twenty-one (51%) FPs at PCN A utilized ABCp by referring at least one patient to the pathway. Notably, the first referrals to ABCp were sent in December 2021, nearly eight months after FPs from PCN A enrolled in the study in April 2021. Further, of the FPs utilizing the ABCp, the median patient referrals were three (Q1, Q3 = 1, 7). At PCN B, 21/26 (81%) FPs adopted the ABCp. After the initial implementation processes at this PCN, the first referrals to ABCp occurred five months after FP enrolment commenced in May 2021. Of the FPs who adopted the pathway, the median patient referrals to the ABCp was four (Q1, Q3 = 2, 6).

#### Implementation

**Fig 4:**
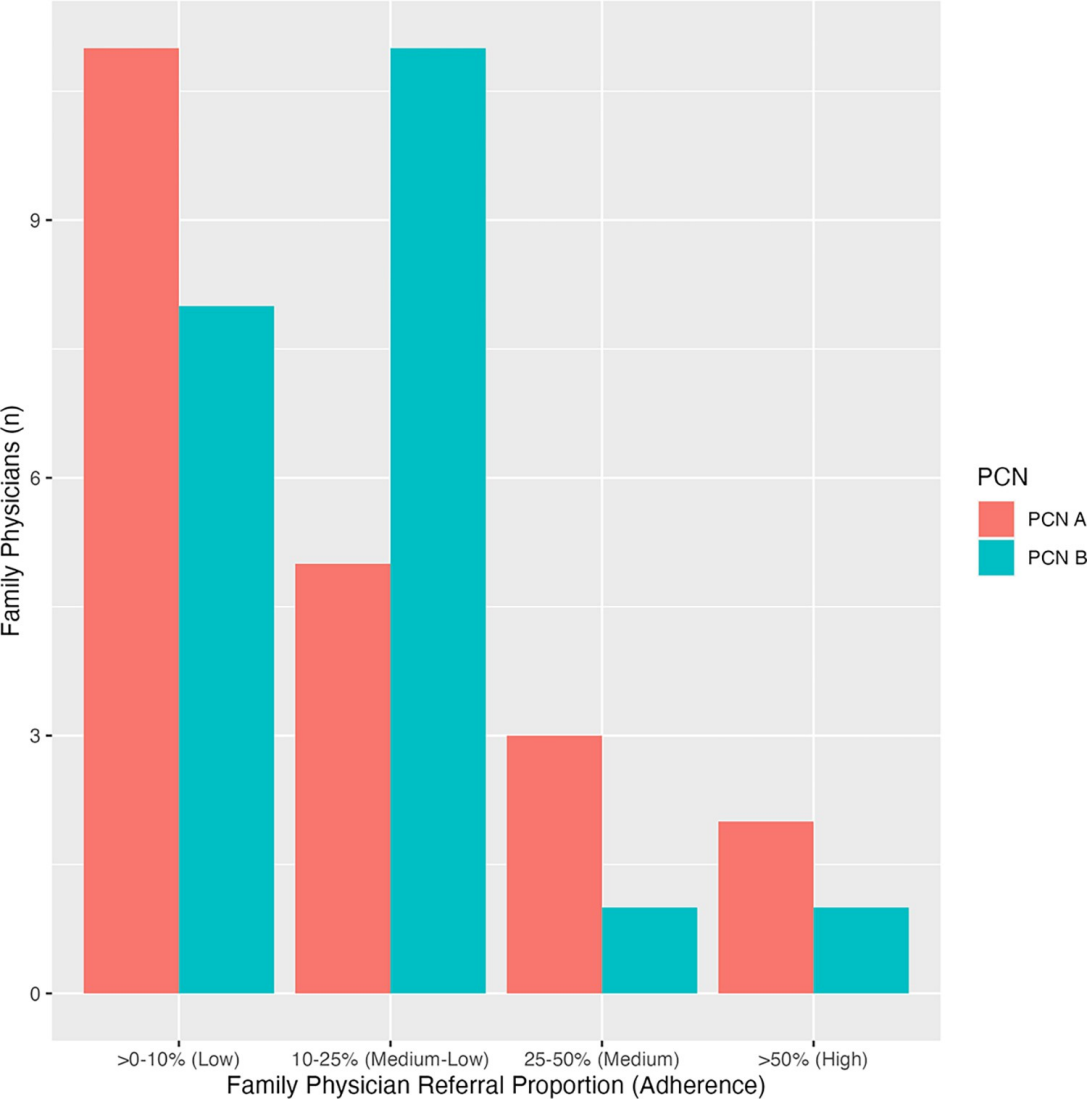
Family physician adherence to the ABCp.

At PCN A, the median FP referral proportion of patients to the pathway from their LBP caseload was 7% (Q1, Q3 = 3, 21). When reporting the FP referral categorically, most FPs 11/21 (52%) had low adherence (>0–10%) in using ABCp with their LBP patients. Comparatively, the median FP referral proportion of LBP patients at PCN B was higher at 13% (Q1, Q3 = 4, 18). When examining the FP referral proportion categorically, there is a slight difference with most FPs 11/21 (52%) having a medium-low adherence (10–25%) to using ABCp with their LBP patients (Fig 4).

#### Maintenance

Over the one year of follow-up, at PCN A, 14/21 (67%) of ABCp FPs continued to refer patients three months after they referred their first patient to the pathway. A small decline in FP maintenance of ABCp was observed with referrals at six months, 10/21 (48%) and at one year, 10/18 (56%). At PCN B, of the FPs that referred to ABCp, we observed sustained pathway use by 14/21 (67%) FPs at three months. As in the first PCN, there was a decline in FP pathway maintenance at six months, 9/21 (43%) but a greater decrease at one year, 7/18 (39%).

### Plan-Do-Study-Act adaptations of implementation

During the course of the study, our Plan-Do-Study-Act process led to specific adaptations to our implementation strategy. Specifically, completion of the training program was initially required before an FP could refer their patients to the ABCp. The Plan-Do-Study-Act approach identified that the training course was too long or too demanding on their time. As a result, mandatory completion of the training program was changed to voluntary completion on January 11, 2022, with FPs having 24/7 access to training materials. With this adaptation, FPs could begin to use the pathway and refer patients as soon as they enrolled in the study. Presently, each FP can now choose the level of training they find most convenient.

### Adverse events

No adverse events or unintended effects were reported.

## Discussion

### Main findings

Implementing the ABCp in two PCNs during the COVID-19 pandemic was feasible. Using the RE-AIM framework, PCN A and B yielded similar implementation feasibility results. Overall, FP enrolment in the study was low, but for those FPs who enrolled, usage of the pathway fulfilled our predefined success criteria (>50%). The proportion of referrals to the ABCp for the majority of those FPs enrolled in the study was below 25% of their caseload, although the number of appropriate referrals in a caseload could not be determined. More than 90% of referrals being made for patients designated as chronic LBP and could, therefore, access the full GLA:D Back program of patient education and supervised exercises. In general, use of the pathway declined over the 12-month follow-up period.

For the majority of patients seeking care for LBP in two urban PCNs, implementing the ABCp did not change usual care from FPs. The 25% and 12% reach among potential FPs at PCN A and PNC B may have resulted from an absence of awareness of the program or the potential benefits of ABCp. A lack of time from the potential FPs, exacerbated by the COVID-19 pandemic, may have also contributed to the failure to implement a new clinical pathway. This possibility is supported by the Canadian Medical Association 2021 Physician Health Survey [40]. The online national survey found that 51% of physician respondents reported experiencing burn out in 2021 (an increase of 1.5x compared to 2017) and reported their top barrier to maintaining their health and wellness was lack of time (64%) and heavy/ stressful workload (60%) [40].

From a system perspective, the structure of how each PCN is managed and the individual clinic priorities, like staffing distribution and programs offered, may also have played a role in the reach of the pathway. Specifically, even though an FP is a member of a PCN, they are independent practitioners and have the final decision to choose the programs that work best for their own clinical practice. In addition, PCN clinical priorities were affected during COVID-19 as evidenced by changing public health priories (like vaccinations and infection surveillance) and increasing needs to care for vulnerable patient populations throughout the pandemic [41].

ABCp adoption by enrolled FP was >50% at both PCNs. These data suggest that those who enrolled in ABCp had a need for such a program and were willing to try the pathway. Although this suggests a high need for a pathway like ABCp, it was not used in its entirety. At both PCN A and PCN B, the majority of referrals to ABCp were chronic LBP patients: 93% and 88%, respectively. This may suggest that FPs have limited resources and time to manage chronic LBP cases properly and therefore found using a fully funded pathway valuable for managing those patients. This is supported by a scarcity of resources felt by FP graduates that have expressed a want for more opportunities and provincial government funding to access interprofessional, collaborative, team-based care [42].

Reasons for low to medium-low FP referral proportions of their LBP patients to ABCp is likely multifactorial. For example, patients may have been offered to be referred to the pathway by their FP but declined to enroll. Similarly, it may be more convenient for some FPs or patients to give/receive usual care by an FP rather than refer/obtain care elsewhere. A Canadian LBP care pathway previously introduced also demonstrated similar barriers during the early stages of implementation, where enrolled FPs continued to refer patients in the conventional or usual ways to specialists instead of using the clinical care pathway [21].

Maintenance of ABCp was high among FPs in the short term, which indicates that once the FPs were exposed to the pathway and its benefits, they found it worthwhile, although their use of the ABCp declined at six and 12 months in both PCNs. This trend could be due to normal study attrition, which is common in most scientific trials [43], or the needs of FPs changed during the course of the study. The observed decline in the ABCp usage may also be attributed to the lack of a consistently sustained support strategy by the research team with regard to frequency and/or duration of implementation interventions, both of which have been shown to play an important role in successful implementation [44].

Last, FP engagement was challenging throughout the study. The 17% completion rate at PCN A and 23% at PCN B of the 4-month survey was consistent with other signals obtained from the FPs during this study, such as limited time to enroll, perform training, and attend meetings. Communications from the PCNs suggested these difficulties were experienced by other new programs attempting to be implemented during the pandemic–a time when FPs were known to struggle with workload and other stressors [40,45]. Non pandemic clinician implementation facilitators/barriers could be similar to what was observed with GLA:D Back in Denmark, where implementors of the program had more positive beliefs on factors such as personal gain, practicalities, and buying-in to the program’s cognitive behavioral approach to LBP care compared to non-implementors [46]. Further exploration of the facilitators and barriers at all participant levels of implementing the ABCp at PCNs is needed to understand the implementation outcomes. As a result, and without sufficient data from the follow-up survey, we are left to speculate about several of our feasibility outcomes.

### Study strengths and limitations

Overall, in this feasibility study the implementation strategy was well planned, including the use of theoretical frameworks (QIF, RE-AIM and PDSA) to help guide, evaluate, and understand the implementation process and the development of a high-quality online training course to help support the understanding of ABCp and its use in practice.

The COVID-19 pandemic was a significant factor in the ABCp implementation. In Canada, the COVID-19 pandemic was declared a national state of emergency on March 23, 2020, with subsequent spikes in hospitalizations due to COVID-19 in December 2020, April 2021, September 2021 and January 2022 [47]. Given that implementation of ABCp began in April 2021 at PCN A, it is highly likely FPs’ priorities were not aligned with enrolling in new studies or programs. However, testing the feasibility of the ABCp in PCNs during the COVID-19 pandemic must also be viewed as a strength in this study because it demonstrates that it can be feasible to implement this LBP pathway when barriers are likely the highest they will be (i.e., during a global pandemic). Testing the feasibility of the ABCp at two similar PCNs was a strength of this study as it allowed us to understand that implementation barriers observed from assessing the dimensions in the RE-AIM framework at one PCN was not just an outlier. Similar implementation outcomes were also observed at the second PCN (reach being <50%, low patient referral rates and decline in usage of the pathway over 12 months).

Reasons for low uptake, that are most likely not all related to the COVID-19 pandemic, were not investigated; however, a future qualitative study at both participation levels would help us explore these factors. Given that the 4-month follow-up survey response rates were so low at both PCNs, data analysis was not justifiable for the outcomes like the DIBQ, PCS and PABS. Last, an assessment of the fidelity to determine if the pathway and intervention program was delivered the way it was intended will be relevant when the ABCp is implemented at a more substantial capacity and as such was not evaluated. This study did not report the effectiveness of the intervention in the ABCp, which will be reported in a future paper.

### Recommendations for future studies

Qualitative methods to further define ABCp barriers and facilitators would provide critical insights at the FP level. For example, understanding FPs’ low interest in the pathway, low motivation for training and if other formats are required, their preferences, and need to find such a care pathway valuable and worthwhile is essential. Understanding the patient’s perspective of the ABCp would also be valuable because it would generate evidence of whether the pathway was helpful in managing their LBP. In addition, continued study of ABCp implementation when the impact of COVID-19 is less severe would be fundamental toward the spread and scale of this pathway (or similar ones) across more PCNs in multiple health settings, both urban and rural, and health systems, such as different provinces in Canada. Future studies must be designed to investigate if changing pathways can reduce the inequality in care for LBP, which was not investigated in this study.

## Conclusion

The ABCp, used by FPs, was feasible to implement at two large urban PCNs in Alberta during a global pandemic, but few utilized the pathway to its full capacity. Most FPs referred patients with chronic LBP to the pathway to receive a supervised, guideline-based education and exercise group program. Research is urgently needed to understand the barriers and facilitators of implementing evidence-based care for LBP in primary care.

## Supporting information

S1 Checklist TREND statement checklist. (PDF)

S1 File (DOCX)

## References

[1] Hoy D, Bain C, Williams G, March L, Brooks P, Blyth F, et al. A systematic review of the global prevalence of low back pain. Arthritis and Rheumatism. 2012. pp. 2028–2037. doi: 10.1002/art.34347 22231424

[2] Vos T, Abajobir AA, Abate KH, Abbafati C, Abbas KM, Abd-Allah F, et al. Global, regional, and national incidence, prevalence, and years lived with disability for 328 diseases and injuries for 195 countries, 1990–2016: a systematic analysis for the Global Burden of Disease Study 2016. Lancet. 2017;390: 1211–1259. doi: 10.1016/S0140-6736(17)32154-2 28919117 PMC5605509

[3] Hartvigsen J, Hancock MJ, Kongsted A, Louw Q, Ferreira ML, Genevay S, et al. What low back pain is and why we need to pay attention. Lancet. 2018;391: 2356–2367. doi: 10.1016/S0140-6736(18)30480-X 29573870

[4] Fatoye F, Gebrye T, Ryan CG, Useh U, Mbada C. Global and regional estimates of clinical and economic burden of low back pain in high-income countries: a systematic review and meta-analysis. Front Public Health. 2023;11: 1098100. doi: 10.3389/fpubh.2023.1098100 37383269 PMC10298167

[5] Hoy D, March L, Brooks P, Blyth F, Woolf A, Bain C, et al. The global burden of low back pain: estimates from the Global Burden of Disease 2010 study. Ann Rheum Dis. 2014;73: 968–974. doi: 10.1136/annrheumdis-2013-204428 24665116

[6] Gross DP, Ferrari R, Russell AS, Battié MC, Schopflocher D, Hu RW, et al. A population-based survey of back pain beliefs in Canada. Spine. 2006;31: 2142–2145. 16915103 10.1097/01.brs.0000231771.14965.e4

[7] Qaseem A, Wilt TJ, McLean RM, Forciea MA, Clinical Guidelines Committee of the American College of Physicians, Denberg TD, et al. Noninvasive Treatments for Acute, Subacute, and Chronic Low Back Pain: A Clinical Practice Guideline From the American College of Physicians. Ann Intern Med. 2017;166: 514–530. doi: 10.7326/M16-2367 28192789

[8] Devereaux MW. Neck and low back pain. Med Clin North Am. 2003;87: 643–662. doi: 10.1016/s0025-7125(03)00002-6 12812407

[9] WHO guideline for non-surgical management of chronic primary low back pain in adults in primary and community care settings. World Health Organization; 7 Dec 2023 [cited 18 Dec 2023]. Available: https://www.who.int/publications/i/item/9789240081789. 38198579

[10] Stochkendahl MJ, Kjaer P, Hartvigsen J, Kongsted A, Aaboe J, Andersen M, et al. National Clinical Guidelines for non-surgical treatment of patients with recent onset low back pain or lumbar radiculopathy. European Spine Journal. 2018. pp. 60–75. doi: 10.1007/s00586-017-5099-2 28429142

[11] National Guideline Centre (UK). Low Back Pain and Sciatica in Over 16s: Assessment and Management. London: National Institute for Health and Care Excellence (NICE); 2016. 27929617

[12] Toward Optimized Practice. Evidence-informed primary care management of low back pain. 2015 [cited 10 Jun 2022]. Available: https://actt.albertadoctors.org/CPGs/Lists/CPGDocumentList/LBP-guideline.pdf.

[13] Kirkwood J, Am C, Allan GM, Christina C, Ccfp SK, Mccormack J, et al. PEER simplified decision aid: chronic back pain treatment options in primary care. Canadian Family Physician. 2021;67: 31–34. doi: 10.46747/cfp.670131 33483394 PMC7822602

[14] Williams CM, Maher CG, Hancock MJ, McAuley JH, McLachlan AJ, Britt H, et al. Low back pain and best practice care: A survey of general practice physicians. Arch Intern Med. 2010;170: 271–277. doi: 10.1001/archinternmed.2009.507 20142573

[15] Sabesan VJ, Schrotenboer A, Habeck J, Lombardo D, Stine S, Jildeh TR, et al. Musculoskeletal education in medical schools: A survey of allopathic and osteopathic medical students. J Am Acad Orthop Surg Glob Res Rev. 2018;2: e019. doi: 10.5435/JAAOSGlobal-D-18-00019 30211396 PMC6132304

[16] Mansfield CD. Attitudes and behaviours towards clinical guidelines: the clinicians’ perspective. Qual Health Care. 1995;4: 250–255. 10156394 10.1136/qshc.4.4.250 PMC1055335

[17] Fischer F, Lange K, Klose K, Greiner W, Kraemer A. Barriers and strategies in guideline implementation-A scoping review. Healthcare (Basel). 2016;4: 36. doi: 10.3390/healthcare4030036 27417624 PMC5041037

[18] Dixon-Woods M, McNicol S, Martin G. Ten challenges in improving quality in healthcare: lessons from the Health Foundation’s programme evaluations and relevant literature. BMJ Qual Saf. 2012;21: 876–884. doi: 10.1136/bmjqs-2011-000760 22543475 PMC3461644

[19] Panella M, Vanhaecht K. Is there still need for confusion about pathways? International Journal of Care Pathways. 2010;14: 1–3.

[20] Fourney DR, Dettori JR, Hall H, Härtl R, McGirt MJ, Daubs MD. A systematic review of clinical pathways for lower back pain and introduction of the Saskatchewan Spine Pathway. Spine. 2011;36: S164–71. doi: 10.1097/BRS.0b013e31822ef58f 21952187

[21] Wilgenbusch CS, Wu AS, Fourney DR. Triage of spine surgery referrals through a multidisciplinary care pathway: a value-based comparison with conventional referral processes. Spine. 2014;39: S129–35. doi: 10.1097/BRS.0000000000000574 25299256

[22] Zarrabian M, Bidos A, Fanti C, Young B, Drew B, Puskas D, et al. Improving spine surgical access, appropriateness and efficiency in metropolitan, urban and rural settings. Can J Surg. 2017;60: 342–348. doi: 10.1503/cjs.016116 30246685 PMC5608584

[23] Health Canada = Santé Canada. An Action Plan for Pain in Canada. Health Canada = Santé Canada; 2021. Available: https://www.canada.ca/en/health-canada/corporate/about-health-canada/public-engagement/external-advisory-bodies/canadian-pain-task-force/report-2021.html.

[24] Curran GM, Bauer M, Mittman B, Pyne JM, Stetler C. Effectiveness-implementation hybrid designs: combining elements of clinical effectiveness and implementation research to enhance public health impact. Med Care. 2012;50: 217–226. doi: 10.1097/MLR.0b013e3182408812 22310560 PMC3731143

[25] Kjaer P, Kongsted A, Ris I, Abbott A, Rasmussen CDN, Roos EM, et al. GLA:D ® Back group-based patient education integrated with exercises to support self-management of back pain—development, theories and scientific evidence. BMC Musculoskelet Disord. 2018;19: 1–21. 30497440 10.1186/s12891-018-2334-x PMC6267880

[26] Kongsted A, Hartvigsen J, Boyle E, Ris I, Kjaer P, Thomassen L, et al. GLA:D® Back: group-based patient education integrated with exercises to support self-management of persistent back pain—feasibility of implementing standardised care by a course for clinicians. Pilot and Feasibility Studies. 2019;5: 1–16. 31086676 10.1186/s40814-019-0448-z PMC6507160

[27] Kongsted A. (Department of Sports Science and Clinical Biomechanics, Odense, Denmark). Conversation with: Brandyn Powelske (Department of Physical Therapy, University of Alberta, Edmonton, Canada). 2024.

[28] Ris Hartvigsen, Kjær Kongsted. GLAD Denmark annual report 2020. SDU; 2020. Available: https://www.glaid.dk/pdf/GLAD_Denmark_annual_report_2020_f.pdf.

[29] Lemieux J, Kawchuk G, Kongsted A, Hartvigsen J, Abdollah V, Jones A. The feasibility of implementing an English language version of GLA:D Back. Pilot Feasibility Stud. 2021;7: 38. doi: 10.1186/s40814-020-00758-z 33522956 PMC7849100

[30] Fernandez M, Young A, Kongsted A, Hartvigsen J, Barton C, Wallis J, et al. GLA:D® Back Australia: a mixed methods feasibility study for implementation. Chiropr Man Therap. 2022;30: 17. 10.1186/s12998-022-00427-3 PMC8989099 35392935

[31] Kongsted A, Ris I, Kjaer P, Vach W, Morsø L, Hartvigsen J. GLA:D® Back: Implementation of group-based patient education integrated with exercises to support self-management of back pain-protocol for a hybrid effectiveness-implementation study. BMC Musculoskelet Disord. 2019;20: 1–21. 30777049 10.1186/s12891-019-2443-1 PMC6380042

[32] Meyers DC, Durlak JA, Wandersman A. The quality implementation framework: a synthesis of critical steps in the implementation process. Am J Community Psychol. 2012;50: 462–480. doi: 10.1007/s10464-012-9522-x 22644083

[33] Bush T, Cherkin D, Barlow W. The impact of physician attitudes on patient satisfaction with care for low back pain. Arch Fam Med. 1993;2: 301–305. doi: 10.1001/archfami.2.3.301 8252151

[34] Ostelo RWJG, Stomp-van den Berg SGM, Vlaeyen JWS, Wolters PMJC, de Vet HCW. Health care provider’s attitudes and beliefs towards chronic low back pain: the development of a questionnaire. Man Ther. 2003;8: 214–222. doi: 10.1016/s1356-689x(03)00013-4 14559044

[35] Atkins L, Francis J, Islam R, O’Connor D, Patey A, Ivers N, et al. A guide to using the Theoretical Domains Framework of behaviour change to investigate implementation problems. Implement Sci. 2017;12: 77. doi: 10.1186/s13012-017-0605-9 28637486 PMC5480145

[36] Schröder K, Öberg B, Enthoven P, Kongsted A, Abbott A. Confidence, attitudes, beliefs and determinants of implementation behaviours among physiotherapists towards clinical management of low back pain before and after implementation of the BetterBack model of care. BMC Health Serv Res. 2020;20: 443. doi: 10.1186/s12913-020-05197-3 32430047 PMC7238530

[37] Glasgow RE, Vogt TM, Boles SM. Evaluating the public health impact of health promotion interventions: the RE-AIM framework. Am J Public Health. 1999;89: 1322–1327. doi: 10.2105/ajph.89.9.1322 10474547 PMC1508772

[38] Glasgow RE, Estabrooks PE. Pragmatic applications of RE-AIM for health care initiatives in community and clinical settings. Prev Chronic Dis. 2018;15: 1–7. doi: 10.5888/pcd15.170271 29300695 PMC5757385

[39] RE-AIM–Home–Reach Effectiveness Adoption Implementation Maintenance. [cited 24 Jan 2023]. Available: https://re-aim.org/.

[40] Canadian Medical Association. CMA 2021 National Physician Health Survey. 2022 Aug.

[41] Mathews M, Meredith L, Ryan D, Hedden L, Lukewich J, Marshall EG, et al. The roles of family physicians during a pandemic. Healthc Manage Forum. 2023;36: 30–35. doi: 10.1177/08404704221112311 35848444 PMC9297067

[42] Sirianni G, Xu QYA. Canada’s crisis of primary care access: Is expanding residency training to 3 years a solution? CMAJ. 2023;195: E1418–E1419. doi: 10.1503/cmaj.230561 37871947 PMC10593194

[43] Dumville JC, Torgerson DJ, Hewitt CE. Reporting attrition in randomised controlled trials. BMJ. 2006;332: 969–971. doi: 10.1136/bmj.332.7547.969 16627519 PMC1444839

[44] Mesner SA, Foster NE, French SD. Implementation interventions to improve the management of non-specific low back pain: a systematic review. BMC Musculoskelet Disord. 2016;17. doi: 10.1186/s12891-016-1110-z 27286812 PMC4902903

[45] Shanafelt TD, West CP, Dyrbye LN, Trockel M, Tutty M, Wang H, et al. Changes in Burnout and Satisfaction With Work-Life Integration in Physicians During the First 2 Years of the COVID-19 Pandemic. Mayo Clin Proc. 2022;97: 2248–2258. doi: 10.1016/j.mayocp.2022.09.002 36229269 PMC9472795

[46] Ris I, Boyle E, Myburgh C, Hartvigsen J, Thomassen L, Kongsted A. Factors influencing implementation of the GLA:D Back, an educational/exercise intervention for low back pain: a mixed-methods study. JBI Evid Implement. 2021;19: 394–408. doi: 10.1097/XEB.0000000000000284 33965996 PMC8635265

[47] Respiratory virus dashboard. [cited 19 Dec 2023]. Available: https://www.alberta.ca/stats/dashboard/respiratory-virus-dashboard.htm?data=severe-outcomes.

