# Supplementary material for: The Alberta Back Care Pathway: The feasibility of implementing a novel care pathway to improve low back pain management for family physicians in primary care
Source: PLoS One. 2024 Nov 27;19(11):e0312737. doi: 10.1371/journal.pone.0312737 (PMC11602090; doi:10.1371/journal.pone.0312737)
Supplement: S1 File — (DOCX) [file pone.0312737.s002.docx]

**Alberta Back Care Pathway**

Proposed protocol for UAlberta

November 3, 2020

**Purpose**:

To evaluate the feasibility of a funded, integrated pathway for low back pain in Alberta, Canada.

**Justification:**

Low back pain (LBP) is a global problem creating more disability than cardiovascular disease and cancer.

In Canada, many people with LBP seek care from general practitioners (GPs) whose services are fully funded by public health programs. Although “free” to Canadians, most approaches used by physicians to address LBP are of low-value – they are often ineffective and potentially dangerous. While research shows that effective LBP interventions are currently available including education, therapeutic exercise, reassurance, manual therapy and non-opioid medications, most LBP patients don’t receive these more effective approaches because of their cost (they are not fully funded by public health care) and their GPs are not trained to deliver them. As a result, most LBP patients receive ineffective care that creates 1) unnecessary pain, ongoing disability and potential painkiller addiction, 2) misuse of limited health resources and 3) systemic de-integration of community clinicians who provide evidence-based care for LBP, but are not funded by the current health care system (e.g. Chiropractors and Physical Therapists). To overcome the increasing provision of low-value care for LBP in Canada, we have created the Alberta Back Care pathway (ABCp). The ABCp incorporates GLA:D Back programming (Good Life with Osteoarthritis: Denmark) the elements of which include patient education, structured exercise and data collection. This new program provides evidence-based care at no cost to the patient through innovative decision-making tools that de-medicalize LBP care.

**Objectives:**

Our first objective is to assess the feasibility of delivering a novel care pathway designed to shift health resource utilization of LBP patients from low-value to high-value care in both primary and secondary care settings. Our secondary objectives are related to health resource utilization (as measured by cost reduction in GP LBP visits, GP related LBP imaging and GP requested specialist consultations) and patient-reported outcomes (as measured by self-reported back pain, disability and self-efficacy).

**Hypotheses**:

Hypothesis 1: The ABCp will be successfully implemented and feasible (at least 75% of eligible patients enrolled) at both the Edmonton West Primary Care Network (EWPCN) and the Alberta Health Services (AHS) Chronic Pain Centre (CPC) in Calgary. Hypothesis 2: The healthcare resource utilization costs, at both sites, will decrease (a minimal 30% reduction in revisits, imaging and referrals). We expect the potential savings from ABCp use will enable the EWPCN and the CPC to sustain its continued provision into the future.

**Background:**

LBP is a global problem responsible for more years lived with disability than any other condition[1, 2]. As a result, health, societal, and economic burdens associated with LBP approach those of cardiovascular disease, cancer, mental illness, and autoimmune diseases [3].

While current evidence-based guidelines consistently recommend education, therapeutic exercise and

non-opioid medications [4–6], these recommendations are infrequently delivered by GPs for many

reasons [7, 8] including a lack of incentives [9, 10] and lack of effective implementation strategies [11–

13]. In addition, patients often have preferences for imaging, procedures and referrals and

GPs or other clinicians may be complicit in providing ineffective recommendations to appease their patients [14–16]. Even when first-line treatments are available, they typically require out-of-pocket payment or a health benefit plan thereby severely limiting access for many Canadians. Together, these factors have conspired globally to significantly increase chronic LBP while also increasing use of narcotics, imaging, and specialist referrals [17].

The ~700,000 annual healthcare visits in Alberta related to LBP illustrate the above problems clearly.

Guideline-discordant LBP care accounts for much of the ~$54,000,000 spent on Emergency Departments

(ED), Primary Care Networks (PCN) and specialist visits [18]. Guideline-discordant LBP care is not only low value, but is potentially harmful [1, 19, 20]. Up to 22% of persons with LBP in Alberta manage their

condition with opioids [21], creating significant risk for opioid use disorder. Potential harms also arise

from care delays. Up to 90% of LBP surgical referrals in Alberta never go to surgery [22] which creates

consultation wait times of up to 2 years as well as chronicity or additional disability for LBP patients who

could benefit from non-invasive interventions. Further, up to 50% of spine imaging is unnecessary [23]

which can create nocebo messaging that can initiate or perpetuate care-seeking behavior [24].

First, this study will measure the feasibility of implementation of the ABCp into clinical practice at the sites. Implementation will be measured by the number of eligible patients enrolled and qualitative data obtained from clinicians and patients through interviews and surveys.

Second, this study will measure health care utilization. This will be measured through system costs: patient visits/revisits, specialist referrals and spinal imaging. Patient outcomes will also be measured: back pain, disability, and self efficacy.

**Study design**:

A full-scale feasibility trial utilizing a cluster-randomized design in a primary care setting (EWPCN) as well as a secondary care setting (CPC).

**METHODS**

**Overall timeline and setting:**

Our first 1-6 months (interim stage) will consist of project staging and pre-implementation activities. A

project charter consisting of terms of reference for team expectations, accountability and conflict

resolution will be completed and ratified by the leadership team and patient advocates. Project

infrastructure will be established including approved ethics protocols, operational approvals, REDCap

design and testing, creation of a web presence and designing infographics. Following the interim period,

clinician training will commence for EWPCN GPs and CPC GLA:D Back clinicians. Training materials will be

presented in a form that best suits the team structure and may consist of educational sessions, team

seminars, video vignettes, and one-on-one meetings. Frontline staff training will consist of a web-based video that can be accessed at the staff’s convenience.

With completion of pre-implementation activities, full project implementation will begin. Our timeline is

Illustrated and attached in the documentation section. The commencement date of enrollment will be flexible to accommodate COVID-19 delays if present (November 2020 – January 2021) and we are now able to provide GLA:D Back on a telehealth platform if needed. Enrollment will occur for a period of one year. Once enrollment ends, a one-year follow up period will begin. At completion of enrollment, we will be able to obtain health resource utilization data from the AHS Enterprise Data Warehouse for each participant during the 9 month period before they enrolled in the study. Data related to patient reported outcome measures (PROMs) are not collected by Government of Alberta and therefore, only health resource utilization can be compared prior to enrollment during the trial and in the year follow-up period. Both secondary outcomes (health resource utilization and PROMs) will be compared to traditional concurrent controls (usual care). In total, the duration of the project will be 2.5 years or 30 months (1-6 months pre-implementation activities, 12 months of enrollment, 12 months follow up).

**Recruitment:**

The EWPCN leadership will identify interested clinics in their jurisdiction and interested clinicians from those clinics will enroll in the study to a maximum of 50% of all clinicians in the PCN.

The CPC leadership will be responsible for enrolling low back pain referrals into ABCp. Those not enrolled will serve as controls.

**Inclusion / Exclusion criteria:**

Persons having LBP will be defined as having pain localized between the costal margins and the inferior gluteal folds, with or without leg pain (i.e. sciatica) not caused by nonmusculoskeletal or red flag conditions. Those excluded from the study will not meet the above definition or have LBP subsequently diagnosed to arise from a non-spinal condition (e.g. renal colic). The definition will also therefore exclude those individuals with pain radiating to the lower back. This includes any hip, pelvic pain which is the primary origin. Ex: hip osteoarthritis, labral lesions, or myofascial pain complaints eg trochanteric bursitis/iliotibial band tension/piriformis syndrome and pelvic floor pain.

*Patients* will be age ≥18 years; (no upper age limit), English speaking and a resident of Alberta. GLA:D Back has been designed in part using clinical practice guidelines that were constructed for adults. In the community, GLA:D Back does not enroll those who are < 18 years of age. Taken together, only those who are 18 years or older will be considered for enrollment. GLA:D Back materials for participants and clinicians have been prepared in English only. As such, only those able to communicate in English will be enrolled.

*Cognition and language sufficient to understand written information, and to provide consent.* In this study, participants require a Grade 4 reading comprehension level so that they can evaluate consent materials.

*People with persistent and/or recurrent LBP with a need for improved self- management.* In the community, GLA:D Back has been designed for persistent low back pain. This does not include acute low back pain or recent onset back pain each of which may be self-limiting within the GLA:D Back intervention period (10 weeks). This can include persons with referred leg pain (but not those with nerve root involvement). Specifically, GLA:D Back pilot data have reinforced these criteria; those who benefit most from GLA:D Back are those who have persistent pain when defined as those currently having low back pain that has impacted daily activities for >1 month. Participants are not eligible to take part in this research study if they are pregnant or suspect they are pregnant.

*Participating currently in a supervised exercise or rehabilitation program or enrolled in another interventional clinical trial.* This requirement is designed to prevent contamination or confounding of our study by requirements of other studies.

*Patients with nerve root involvement, suspected serious pathology, unstable trauma to the spine, pregnant.* Persons with conditions that require a specialist consult are suspected of needing a specialist consult, or have conditions that would be aggravated or worsened if treated by the interventions in this study, will be excluded. This decision will be made first by the principal investigator, then escalate to include available trial-clinicians, and the Adverse Event advisor as needed. In specific cases where there is leg pain, pain radiating into the leg will be permitted into the study but nerve root involvement is an exclusion.

*Planned spine or other major surgery within the trial period.* Potential participants who are planning on undergoing surgery that would involve the spine directly or indirectly, or place them into a position where any of the interventions in this trial would be contraindicated as a result of the surgery, will be excluded from the study.

*Any known contraindication to exercise (i.e. unstable angina, uncontrolled arrhythmia, severe aortic stenosis).* This criterion is self-explanatory.

For *virtual delivery* of the ABCp (Tele GLA:D Back), patients will need an active connection to the internet and an electronic device with a webcam capable to stream video and audio to the internet (computer, tablet, cellphone) and will have to complete a technology assessment session before the program starts. Patients will also need space to do the exercise in their home and have a yoga mat and TheraBand. If the patient does not have access to any of this equipment, they will be responsible for obtaining these items themselves; otherwise, they will not be eligible for enrollment. See the attached checklist for technical requirements for patients.

For the CPC site specifically, inclusion/exclusion criteria will include the above with the additional requirement of depression and anxiety as rated using the PHQ4 exclusion will include score of 3 or greater on the first two questions combined and/or score of 3 or greater on the third and 4th question combined.

**Consenting:**

Those patients meeting the inclusion and exclusion criteria of ABCp and who provide consent will be enrolled into the study as patients. All consent will be done electronically through REDCap.

**Data Acquisition:**

Feasibility measures will derive data directly from provincial data sources with the exception of qualitative interviews/surveys. For the aggregate healthcare utilization savings, all data is obtained from a singular data source from the province.

For patient reported outcome measures all data will be acquired through REDCap (Research Electronic Data Capture) which is a browser-based, software solution that allows researchers to create secure online forms for data capture, management, and analysis. Clinicians will be sent a link that can access this portal through a secure REDCap login generating data through questionnaires and surveys. In turn, they will provide patients with a secure REDCap login where patient data will also be collected. REDCap will be the application used to manage the outcome questionnaires, surveys and tracking of clinicians and patients enrolled in the GLA:D program.

**Primary Outcomes:**

ABCp feasibility will be assessed by the percentage of eligible patients enrolled in the trial, and qualitative markers from stakeholder interviews/surveys. We expect qualitative outcomes will reflect attitudes for continued support of the pathway by all involved.

**Secondary Outcomes:**

Healthcare utilization cost savings will be measured by reduction in 1) GP revisits for LBP, 2) GP ordered LBP imaging and 3) GP requested specialist consultations. These data will be gathered directly from the AHS Enterprise Data Warehouse.

**Patient level outcomes**:

Collecting of patient reported outcome measures are an integrated part of GLA:D Back and are recorded in REDCap. These measures are described below.

Patient assessments for the GLA:D back program will involve the collection of patient-reported outcome measures (PROMs). PROMs will be collected at the University of Alberta via REDCap. At each assessment, patients will input data using a computer. If necessary, paper forms will be available with data uploaded at a later time.

| Domain | Instrument | Pt. Baseline registration | Post-Program | 3 months post-program | 6 months post-program | 12 months post-program |
| --- | --- | --- | --- | --- | --- | --- |
| Demographics | Self-Report Demographic Data: age, sex, race/ethnicity, employment status, and general medical and LBP history. | x |  |  |  |  |
| Disability and Activity Limitation | Oswestry Disability Index Ten questions scored 0-5 per section. Higher score suggested more disabled | x |  | x | x | x |
| Pain Intensity | Numeric Rating Scale 0-10 for LBP and leg pain | x |  | x | x | x |
| Fear of Movement | Fear Avoidance Beliefs Questionnaire 16 questions (physical activity and work subscales) using a scale from 0, completely disagree, to 6, completely agree for all questions | x |  | x | x | x |
| Tele-GLA:D Back Beliefs and Satisfaction  (technical quality, effect on communication, ease of use, comfort, accessibility, perceived usefulness, perceived effectiveness, intention to use, overall satisfaction) | The Telemedicine Satisfaction Questionnaire using a scale from 1, strongly disagree, to 5, strongly agree for all 14 questions (Yip et al., 2003) |  | x |  |  |  |

Subject Self-Report Measures Demographic Data: Subjects will provide demographic information including age, sex, race/ethnicity, employment status, and general medical and LBP history.

Oswestry Disability Questionnaire (ODQ): The ODQ is a LBP-specific measure of function for patients with LBP assessed on a 0-100 scale, with lower numbers indicating better function. Past research has found the ODQ to have high test-retest reliability (ICC=0.9), good construct validity, and responsiveness to change for patients with LBP, with a minimum clinically important difference of 6 points.

Numeric Pain Rating Scale (NPRS): A 0-10 NPRS (“0” no pain, and “10” worst pain imaginable) will be used to assess LBP intensity. The NPRS has excellent test-retest reliability. Previous research has found the NPRS to be responsive to change, with an MCID of 2 points for patients with acute LBP.

Psychosocial Covariate Measures: The Fear-Avoidance Beliefs Questionnaire (FABQ) will be used to measure patients’ beliefs about how physical activity and work may affect their LBP and perceived risk for reinjury. The FABQ work score is a factor in the clinical prediction rule.

For Tele-GLA:D technical quality, effect on communication, ease of use, comfort, accessibility, perceived usefulness, perceived effectiveness, intention to use, overall satisfaction will be measured using The Telemedicine Satisfaction Questionnaire (Yip et al., 2003) using a scale from 1, strongly disagree, to 5, strongly agree for all 14 questions.

Telemedicine Satisfaction Questionnaire

1. I can easily talk to my Tele-GLA:D provider
2. I can hear my Tele-GLA:D provider clearly
3. My Tele-GLA:D provider is able to understand my health-care condition
4. I can see my Tele-GLA:D provider as if we met in person
5. I do not need assistance while using the system
6. I feel comfortable communicating with my health-care provider
7. I think the healthcare provided via Tele-GLA:D is consistent
8. I obtain better access to health-care services by use of Tele-GLA:D
9. Tele-GLA:D saves me time travelling to hospital or a specialist clinic
10. I do receive adequate attention
11. Tele-GLA:D provides for my health-care need
12. I find Tele-GLA:D an acceptable way to receive health-care services
13. I will use Tele-GLA:D services again
14. Overall, I am satisfied with the quality of service being provided via Tele-GLA:D

*Telemedicine replaced with Tele-GLA:D

**Statistical Analysis:**

Concurrent time series plots for intervention and control sites, pre- and post-intervention, will

display baseline levels and trends, as well as level and trend changes occurring after protocol activation.

If data analysis of secondary outcomes suggests differences between ABCp vs usual care cohorts, we will

perform complexity adjusted analyses.

**Reference list**

1. Hartvigsen J, Hancock MJ, Kongsted A, et al. (2018) What low back pain is and why we need to

pay attention. Lancet (London, England) 391:2356–2367. doi: 10.1016/S0140-6736(18)30480-X

2. Vos T, Abajobir AA, Abate KH, et al. (2017) Global, regional, and national incidence, prevalence,

and years lived with disability for 328 diseases and injuries for 195 countries, 1990–2016: a systematic

analysis for the Global Burden of Disease Study 2016. Lancet 390:1211–1259. doi: 10.1016/S0140-

6736(17)32154-2

3. Maniadakis N, Gray A (2000) The economic burden of back pain in the UK. Pain 84:95–103.

4. (2016) Low back pain and sciatica in over 16s: assessment and management | Guidance and

guidelines | NICE. Natl. Inst. Heal. Care Excell.

5. Stochkendahl MJ, Kjaer P, Hartvigsen J, et al. (2018) National Clinical Guidelines for non-surgical

treatment of patients with recent onset low back pain or lumbar radiculopathy. Eur Spine J 27:60–75. doi:

10.1007/s00586-017-5099-2

6. Wong JJ, Côté P, Sutton DA, et al. (2017) Clinical practice guidelines for the noninvasive

management of low back pain: A systematic review by the Ontario Protocol for Traffic Injury Management (OPTIMa) Collaboration. Eur J Pain 21:201–216. doi: 10.1002/ejp.931

7. Breen A, Austin H, Campion-Smith C, et al. (2007) “You feel so hopeless”: a qualitative study of GP

management of acute back pain. Eur J Pain 11:21–9. doi: 10.1016/j.ejpain.2005.12.006

8. Darlow B, Dean S, Perry M, et al. (2014) Acute low back pain management in general practice:

uncertainty and conflicting certainties. Fam Pract 31:723–732. doi: 10.1093/fampra/cmu051

9. F ischer F, Lange K, Klose K, et al. (2016) Barriers and Strategies in Guideline Implementation-A

Scoping Review. Healthc (Basel, Switzerland) 4:36. doi: 10.3390/healthcare4030036

10. Dixon-Woods M, McNicol S, Martin G (2012) Ten challenges in improving quality in healthcare:

lessons from the Health Foundation’s programme evaluations and relevant literature. BMJ Qual Saf

21:876–84. doi: 10.1136/bmjqs-2011-000760

11. Liang L, Abi Safi J, Gagliardi AR, members of the Guidelines International Network Implementation Working Group (2017) Number and type of guideline implementation tools varies by guideline, clinical condition, country of origin, and type of developer organization: content analysis of guidelines. Implement Sci 12:136. doi: 10.1186/s13012-017-0668-7

12. Mesner SA, Foster NE, French SD (2016) Implementation interventions to improve the

management of non-specific low back pain: a systematic review. BMC Musculoskelet Disord 17:258. doi:

10.1186/s12891-016-1110-z

13. Suman A, Dikkers MF, Schaafsma FG, et al. (2016) Effectiveness of multifaceted implementation

strategies for the implementation of back and neck pain guidelines in health care: a systematic review.

Implement Sci 11:126. doi: 10.1186/s13012-016-0482-7

14. Wilson TJ, Franz E, Vollmer CF, et al. (2017) Patient-perceived surgical indication influences

patient expectations of surgery for degenerative spinal disease. Clin Neurol Neurosurg 157:11–16. doi:

10.1016/j.clineuro.2017.03.007

15. Franz EW, Bentley JN, Yee PPS, et al. (2015) Patient misconceptions concerning lumbar

spondylosis diagnosis and treatment. J Neurosurg Spine 22:496–502. doi: 10.3171/2014.10.SPINE14537

16. Weber C, Behbahani M, Baardsen R, et al. (2017) Patients’ beliefs about diagnosis and treatment

of cervical spondylosis with radiculopathy. Acta Neurochir (Wien) 159:2379–2384. doi: 10.1007/s00701-

017-3356-0

17. Mafi JN, McCarthy EP, Davis RB, Landon BE (2013) Worsening Trends in the Management and

Treatment of Back Pain. JAMA Intern Med 173:1573. doi: 10.1001/jamainternmed.2013.8992

18. MacMillan M. (2019) AHS Data pull 2017-2018.

19. Foster NE, Anema JR, Cherkin D, et al. (2018) Prevention and treatment of low back pain:

evidence, challenges, and promising directions. Lancet (London, England) 391:2368–2383. doi:

10.1016/S0140-6736(18)30489-6

20. Buchbinder R, Tulder M Van, Öberg B, et al. (2018) Viewpoint Low back pain : a call for action.

Lancet 6736:1–5. doi: 10.1016/S0140-6736(18)30488-4

21. Canadian Chiropractic Association (2016) 2016 MSK Report Card Market Research Study.

22. Nataraj A (2019) Personal communication.

23. Emery DJ, Shojania KG, Forster AJ, et al. (2013) Overuse of Magnetic Resonance Imaging. JAMA

Intern Med 173:823. doi: 10.1001/jamainternmed.2013.3804

24. Klinger R, Blasini M, Schmitz J, Colloca L (2017) Nocebo effects in clinical studies. PAIN Reports

2:e586. doi: 10.1097/PR9.0000000000000586
